# Moderate caloric restriction initiated in rodents during adulthood sustains function of the female reproductive axis into advanced chronological age

**DOI:** 10.1111/j.1474-9726.2008.00409.x

**Published:** 2008-10

**Authors:** Kaisa Selesniemi, Ho-Joon Lee, Jonathan L Tilly

**Affiliations:** Vincent Center for Reproductive Biology, Vincent OB/GYN Service, Massachusetts General Hospital/Harvard Medical SchoolBoston, Massachusetts, USA

**Keywords:** aging, caloric restriction, fecundity, fertility, menopause, oocyte, ovary, reproduction

## Abstract

Age-related ovarian failure in women heralds the transition into postmenopausal life, which is characterized by a loss of fertility and increased risk for cardiovascular disease, osteoporosis and cognitive dysfunction. Unfortunately, there are no options available for delaying loss of ovarian function with age in humans. Rodent studies have shown that caloric restriction (CR) can extend female fertile lifespan; however, much of this work initiated CR at weaning, which causes stunted adolescent growth and a delayed onset of sexual maturation. Herein we tested in mice if CR initiated in adulthood could delay reproductive aging. After 4 months of CR, the ovarian follicle reserve was doubled compared to *ad libitum* (AL)-fed age-matched controls, which in mating trials exhibited a loss of fertility by 15.5 months of age. In CR females returned to AL feeding at 15.5 months of age, approximately one-half remained fertile for 6 additional months and one-third continued to deliver offspring through 23 months of age. Notably, fecundity of CR-then-AL-fed females and postnatal offspring survival rates were dramatically improved compared with aging AL-fed controls. For example, between 10 and 23 months of age, only 22% of the 54 offspring delivered by AL-fed females survived. In contrast, over 73% of the 94 pups delivered by 15.5- to 23-month-old CR-then-AL-fed mice survived without any overt complications. These data indicate that in mice adult-onset CR maintains function of the female reproductive axis into advanced age and dramatically improves postnatal survival of offspring delivered by aged females.

## Introduction

Compared to other organ systems, the female reproductive axis ages exceptionally early in life, culminating in a cessation of normal ovarian function that in humans heralds the onset of menopause at around 50 years of age (Richardson *et al*., 1987; Faddy *et al*., 1992). A progressive decline and ultimately exhaustion of the ovarian oocyte-containing follicle reserve is the prime determinant of the age of onset of menopause (Richardson *et al*., 1987; Faddy *et al*., 1992; Tilly, 2001; Charleston *et al*., 2007; Hansen *et al*., 2008), a period in life associated with a loss of fertility and the emergence of a diverse spectrum of health issues resulting from an absence of cyclic ovarian function (Buckler, 2005). Furthermore, concomitant with reduced follicle numbers, the quality of the remaining oocytes in females generally declines with advancing age, leading to extremely poor success rates of natural and assisted fertility attempts (Navot *et al*., 1991; van Kooij *et al*., 1996). This latter outcome is an increasingly problematic issue as more and more women, especially in Western societies, are postponing childbearing to older ages (Ventura, 1989; Ventura *et al*., 2004; Hamilton *et al*., 2007).

While laboratory rodent models do not exhibit menses and, thus, do not undergo a true menopause like humans, female mice do exhibit an age-related decline in their ovarian follicle reserve, leading to a state of natural infertility approximately halfway through their chronological lifespan (Gosden *et al*., 1983; Perez *et al*., 1999; Wu *et al*., 2005). Furthermore, as a consequence of the loss of cyclic ovarian function, aging female mice exhibit many of the same adverse physiological changes observed in postmenopausal women, and have thus been extensively utilized as model for understanding the impact of ovarian failure on the aging female body. For example, past studies have shown that transplantation of young adult mouse ovaries into aging female mice delays the decline in reproductive potential and increases overall life expectancy of the recipient (Cargill *et al*., 2003). More recently, it was reported that minimizing follicle loss and prolonging ovarian function in female mice through targeted inactivation of the pro-apoptotic *Bax* gene (Perez *et al*., 1999) extends fertile lifespan and minimizes many age-related health problems, including bone and muscle loss, excess fat deposition, alopecia, cataracts, deafness, increased anxiety and selective attention deficit (Perez *et al*., 2007). Despite the promising nature of these findings in mice, strategies for delaying ovarian aging or the timing of menopause in women do not currently exist.

Dietary caloric restriction (CR) without malnutrition is known to extend lifespan and minimize age-related dysfunction of many organs, including those of the female reproductive axis, in several animal models (Masoro, 2005). In fact, a beneficial effect of CR on reproductive function in aging rodents has been documented since the early 1900s. In one of the first published studies on this topic, Osborne *et al*. (1917) reported that restricting food intake in young female rats resulted in sustained fertility much later in life. From this, the authors concluded that ‘the menopause has been postponed [by food restriction] long beyond the age at which it usually appears’. Ball *et al*. (1947) showed in mice that restricting caloric intake from weaning until 8 months of age and then allowing the females to *ad libitum* (AL) feed resulted in 13 times more litters over the next 4 months when compared with age-matched continuously AL-fed controls. Litter size as well as regularity of estrous cyclicity of CR-then-AL-fed aging females resembled those of young adult females, collectively indicating that CR can postpone age-related reproductive senescence in mice (Visscher *et al*., 1952; Nelson *et al*., 1985). While the mechanisms by which CR sustains fertility with age remain to be fully characterized, studies with rats and mice have shown that oocyte numbers are higher in CR females when compared to age-matched AL-fed controls (Lintern-Moore & Everitt, 1978; Nelson *et al*., 1985). These findings suggest that the beneficial effects of CR on female reproductive function are at least partly mediated via maintenance of the ovarian follicle reserve in aging animals. Other work has shown that CR also alters the release patterns of many hormones produced by the hypothalamus and pituitary gland, including those involved in the control of ovarian function (reviewed in Martin *et al*., 2008). Hence, the ability of CR to affect reproductive performance probably reflects a very complex process involving modulation of the entire hypothalamic–pituitary–gonadal axis.

Even though several historical studies have reported a beneficial effect of CR on reproductive performance in rodents, there is a large degree of variation in how these experiments were designed (e.g. the age at which CR was initiated and the duration of CR, as well as the severity of the CR relative to total caloric intake in AL-fed controls) and in what outcomes different investigators assessed over the years. Of particular importance, much of the past work that evaluated the effects of CR on long-term fertility in females was derived from studies of CR initiated at weaning, which complicates interpretation of the outcomes due to a retardation of postweaning growth to adulthood and to a delay in the onset of sexual maturation (Merry & Holehan, 1979; Holehan & Merry, 1985; Gonzales *et al*., 2004). Herein, we investigated in female mice the effects of moderate CR initiated in adulthood on reproductive performance with age and offspring survival.

## Results

### Effectiveness of the adult-onset CR protocol

**Fig. 1:**
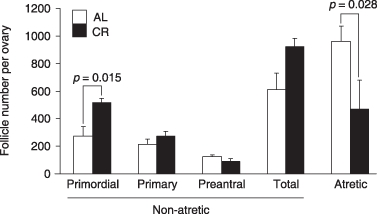
Impact of adult-onset caloric restriction (CR) on the ovarian follicle reserve. Number of non-atretic and atretic immature follicles per ovary in continuously *ad libitum* (AL)-fed or CR female mice at 8 months of age, after initiation of CR 4 months earlier. These data are the mean ± SEM of combined results from analysis of 5 (CR) or 6 (AL-fed) mice, with significant differences in mean values indicated.

Since past studies in mice have shown that one outcome of CR is an increased ovarian follicle reserve later in life (Nelson *et al*., 1985), we first tested the effectiveness of the adult-onset CR protocol by assessing changes in oocyte numbers. Morphometric analysis of healthy (non-atretic) follicle numbers in serially sectioned ovaries of 8-month-old mice indicated that almost twice as many quiescent primordial follicles were present in ovaries of CR females (516 ± 32 per ovary; mean ± SEM, *n* = 5 mice) when compared with ovaries of age-matched continuously AL-fed controls (273 ± 69 per ovary; mean ± SEM, *n* = 6 mice) (Fig. 1). This effect of adult-onset CR appeared to be independent of changes in primordial follicle growth activation rates or early follicle maturation, as indicated by the presence of comparable numbers of small growing follicles and no change in the ratio of resting (primordial) to total immature follicles between experimental groups (Fig. 1). However, the number of atretic immature follicles was reduced in ovaries of adult-onset CR females (468 ± 158 per ovary; mean ± SEM, *n* = 5 mice) when compared to age-matched continuously AL-fed controls (960 ± 112 per ovary; mean ± SEM, *n* = 6 mice).

**Fig. 2:**
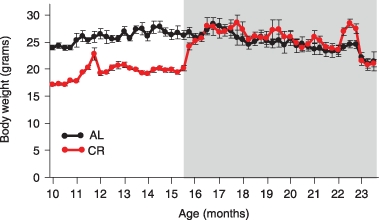
Effect of adult-onset caloric restriction (CR) on body weight. Weekly weight measurements (mean ± SEM) of continuously *ad libitum* (AL)-fed and CR/CR-then-AL-fed female mice between 10 and 23 months of age. Note the return of body weight in CR mice to continuously AL-fed control levels within 2 weeks after resumption of AL-feeding at 15.5 months of age (CR-then-AL-fed), highlighted by grey shading.

The effectiveness of the adult-onset CR protocol was also evaluated by weekly monitoring of body weight, which showed that during restriction CR females weighed approximately 25% less than age-matched continuously AL-fed controls (Fig. 2). Notably, the body weight difference seen in CR females was rapidly reversed after a resumption of AL feeding at 15.5 months of age (Fig. 2).

### Timing of natural reproductive failure in continuously AL-fed females

**Fig. 3:**
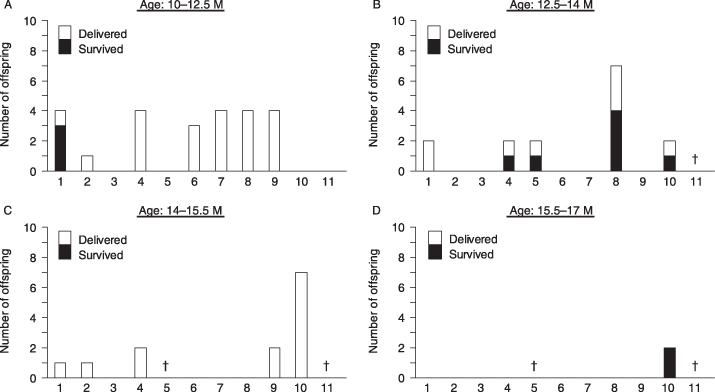
Natural decline in fertility of continuously *ad libitum* (AL)-fed control females with age. (A–D) Fertility of continuously AL-fed females between 10 and 12.5 (A), 12.5 and 14 (B), 14 and 15.5 (C), and 15.5 and 17 (D) months (M) of age (each mouse is represented by a number on the *x*-axis). The total number of offspring delivered and that survived for each female that became pregnant are indicated. Crosses designate mice that had to be euthanized due to severe health complications or that died of natural causes during the study period.

To determine a reference age bracket for the natural decline in reproductive potential of continuously AL-fed females, and thus establish a time point for the re-initiation of AL feeding in the CR cohort, fertility of continuously AL-fed females was monitored in natural mating trials. Between 10 and 12.5 months of age, 7 of 11 continuously AL-fed females became pregnant and delivered offspring (Fig. 3A). Over the following 4.5 months, however, fertility plummeted (Fig. 3B–D), with only a single pregnancy observed in the AL-fed female cohort between 15.5 and 17 months of age (Fig. 3D). Furthermore, offspring survival rates also markedly declined with advancing maternal age of the AL-fed controls (Fig. 3). It should be mentioned here that fertility of CR females while on the restriction protocol (i.e. between 10 and 15.5 months of age) was poor, with a total of 9 pregnancies achieved by the 11 CR females (7 females pregnant once, 1 female pregnant twice). Furthermore, all offspring were delivered dead or died very shortly after birth (data not shown). These outcomes in CR females during the period of food restriction were fully anticipated, however, based on results of both prior studies (Visscher *et al*., 1952) as well as on the logical need for increased energy demands on the pregnant dams not being met under the CR protocol.

### Adult-onset CR prolongs function of the reproductive axis in aging females

**Fig. 4:**
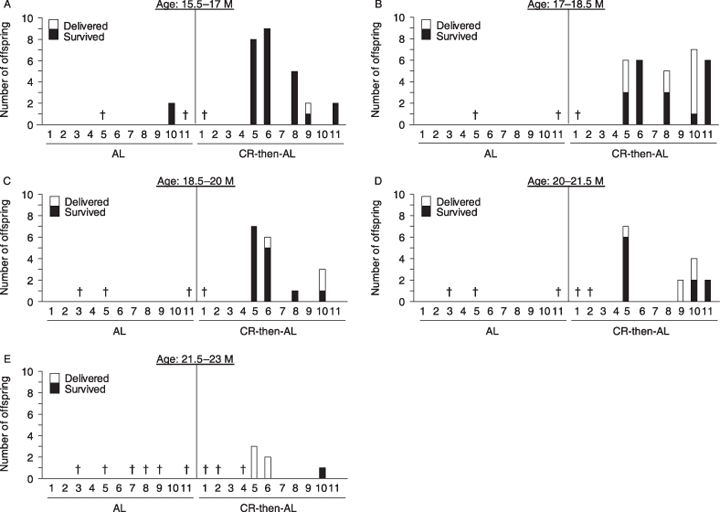
Reproductive performance of aging calorie-restricted (CR) females after re-initiation of *ad libitum* (AL) feeding at 15.5 months of age. (A–E) Fertility outcomes are shown for each continuously AL-fed control (AL) or CR-then-AL-fed female between 15.5 and 17 (A), 17 and 18.5 (B), 18.5 and 20 (C), 20 and 21.5 (D), and 21.5 and 23 (E) months (M) of age (each mouse is represented by a number on the *x*-axis) run in parallel mating trials with the same males randomly rotated among the cages. The total number of offspring delivered and that survived for each female that became pregnant are indicated. Crosses designate mice that had to be euthanized due to severe health complications or that died of natural causes during the study period.

Re-initiation of AL feeding of CR females at 15.5 months of age, which corresponds to a time when natural fertility in the continuously AL-fed controls was lost (Fig. 3), resulted in a rapid return of fertile potential in subsequent mating trials. Specifically, between 15.5 and 17 months of age, 50% of the CR-then-AL-fed females achieved a pregnancy with delivery of offspring (Fig. 4A), and many CR-then-AL-fed females retained reproductive function into more advanced ages (Fig. 4A–E). Litter size as well as offspring survival rates were also remarkably high for CR-then-AL-fed mothers at advanced ages. For example, one 20- to 21.5-month-old CR-then-AL-fed female delivered 7 offspring, of which 6 survived and developed without any apparent complications (female #5, Fig. 4D). Two of the CR-then-AL-fed females actually remained fertile past 23 months of age; however, the 5 total offspring delivered by these females did not survive for more than a few hours after birth (data not shown).

### Fertility, fecundity and offspring survival are all improved by adult-onset CR

**Fig. 5:**
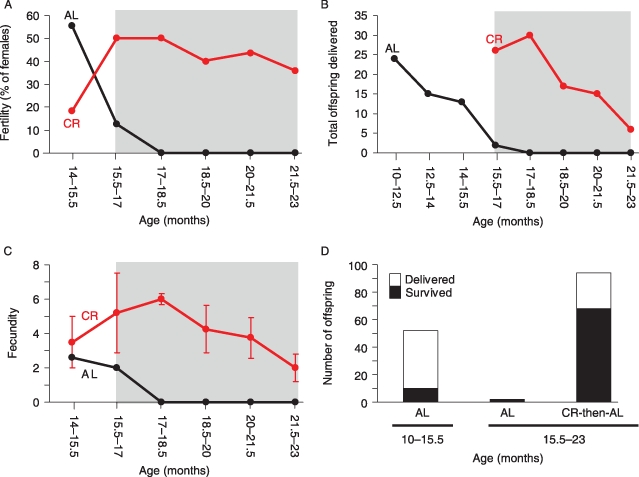
Adult-onset caloric restriction (CR) improves fertility, fecundity and offspring survival rates in aging females. (A–D) Summary of the fertile potential (A, expressed as the percentage of females in each group tested that achieved a pregnancy), total offspring delivered (B), fecundity (C, pups per litter; mean ± SEM), and offspring survival rates (D) in mating trials of continuously AL-fed control females (black lines in A–C) and in CR or CR-then-AL-fed females (red lines in A–C) at the indicated ages. Adult-onset CR females were allowed to resume AL feeding at 15.5 months of age for the remainder of the study period, as highlighted by grey shading in panels A–C. Note the improved outcomes in every endpoint analyzed (A–D) for CR females at each age bracket once AL feeding was resumed.

The data presented in Figs 3 and 4 depict the outcomes for each mouse, so that the reproductive performance of individual females over the course of the mating trial period could be visualized. However, after compiling the individual mouse data, the effects of adult-onset CR on female reproductive performance with age were even more apparent. For example, between 15.5 and 17 months of age, 50% of the CR-then-AL-fed females remained fertile whereas only 12.5% of the continuously AL-fed control females achieved a pregnancy over the same time period (Fig. 5A). Furthermore, 50% of 17- to 18.5-month-old, 40% of 18.5- to 21.5-month-old, and 30% of 21.5- to 23-month-old CR-then-AL-fed females continued to achieve pregnancies with birth of offspring. At these ages, all of the continuously AL-fed females were completely infertile (Fig. 5A).

In evaluating these outcomes from a different perspective, the total number of offspring born to continuously AL-fed females steadily declined between 10 and 17 months of age (Fig. 5B). Strikingly, during an age bracket when only 2 offspring were delivered by a single continuously AL-fed control female (15.5–17 months of age), age-matched CR-then-AL-fed females delivered a total of 25 offspring (Fig. 5B). Similarly, the fecundity (number of offspring per litter) of continuously AL-fed females declined throughout the study period (Fig. 5C) and was consistently below that of age-matched CR-then-AL-fed females. In fact, after re-initiation of AL feeding, the fecundity of previously CR females immediately improved, such that 15.5- to 17-month-old and 17- to 18.5-month-old CR-then-AL-fed females delivered an average of 5.2 ± 2.3 and 6.0 ± 0.3 offspring per litter, respectively (mean ± SEM, *n* = 5 mice per group) (Fig. 5C). Of note, this latter outcome is close to the average litter size of AL-fed C57BL/6 female mice during their peak reproductive period (6.2 pups/litter; see http://www.informatics.jax.org/external/festing/mouse/docs/C57BL.shtml). During the entire mating trial period (10–23 months of age), 9 of the 11 continuously AL-fed females became pregnant and delivered offspring at least once, with a total of 18 pregnancies and 54 offspring delivered. By comparison, between 10 and 23 months of age, 8 of the 11 CR (10–15.5 months)/CR-then-AL-fed (15.5–23 months) females became pregnant and delivered offspring at least once, with a total of 30 pregnancies and 133 offspring delivered. Finally, offspring survival rates were also dramatically improved by adult-onset CR. Specifically, of the 94 total pups delivered by 15.5- to 23-month-old CR-then-AL-fed females, 69 (73.4%) survived and developed normally without any apparent abnormalities (Fig. 5D). In contrast, only 12 of the 54 total pups (22.2%) born to 10- to 17-month-old continuously AL-fed females survived (Fig. 5D; note that no pregnancies were observed in this group past 17 months of age).

In addition, some of the female offspring born to 15.5- to 21.5-month-old CR-then-AL fed females were maintained for up to 12 weeks of age (*n* = 18), and all exhibited normal growth into adulthood with no apparent abnormalities as assessed by gross anatomical examination after euthanasia (data not shown). Normal maturation to adulthood was further indicated by the fertile potential of female offspring (*n* = 3) born to 20- to 21.5-month-old CR-then-AL-fed female mice. After a single mating attempt at 10 weeks of age, these females delivered normal size litters (a total of 19 offspring from the 3 females) with no pup mortality observed (data not shown).

## Discussion

Socioeconomic trends within the past few decades have resulted in more women in developed countries electing to postpone childbearing to later reproductive years. In the USA for example, since the 1970s the rate of first births for women between the ages of 30 and 34 years has increased by 375%. The rate of first births for women older than 35 is even greater, rising within the last four and a half decades by 500% or more for women aged 35–39 and 40–44 years (Ventura, 1989; Ventura *et al*., 2004; Hamilton *et al*., 2007). Perhaps even more striking is the fact that the National Center for Health Statistics (NCHS) has no records of live births for women between 45 and 49 years of age in the 1970s (Ventura, 1989), whereas approximately 7000 live births were documented by the NCHS for mothers within this age range in 2006 (Hamilton *et al*., 2007). Despite this continuously increasing trend to have a first child later in life, when fertility for most women is suboptimal and successful pregnancies may be difficult to achieve even with the aid of modern assisted reproductive technologies, no strategies currently exist to delay the age-related decline in fertility that most women experience in their late 30s and early 40s.

In rodent models, the ability of reduced food intake to postpone age-related infertility in females has been documented for nearly a century (Osborne *et al*., 1917; Ball *et al*., 1947; Visscher *et al*., 1952; Berg, 1960). Although the collective results of these studies are promising, it is important to emphasize that in these earlier studies the CR protocol was often severe and was initiated at weaning. Thus, as normal postnatal growth and development were compromised, the prolongation of female fertile lifespan resulting from CR was attributed to a stunting of overall growth and to a delayed onset of normal sexual maturation (Ball *et al*., 1947). However, this explanation has not been rigorously tested. Given this, herein we tested in mice if age-related failure of the female reproductive axis could be postponed by a moderate adult-onset CR protocol that has recently been shown to effectively maintain overall well-being and increase lifespan of aging rodents (Turturro *et al*., 1999; Masoro, 2005). From these experiments, several important observations were made.

First, in contrast to conclusions reached earlier regarding the mechanism by which CR prolongs ovarian function and fertility (Osborne *et al*., 1917; Ball *et al*., 1947), our studies of aging female mice subjected to an adult-onset CR protocol argue against the idea that CR extends female fertile lifespan by simply stunting adolescent growth and delaying the age at which sexual maturation is reached. In fact, the outcomes obtained herein following the initiation of CR as late as 2 months after attainment of sexual maturity were just as dramatic, if not even more so, as those reported earlier for CR initiated at weaning. For example, over an 8-month period at ages when reproductive capacity had already ceased entirely in continuously AL-fed control females (15.5–23 months of age), many adult-onset CR-then-AL-fed females achieved pregnancies and delivered several litters (up to 5 pregnancies/female) at a rate that was comparable to the fertility observed for continuously AL-fed C57BL/6 female mice during prime reproductive life (for additional details, refer to http://www.informatics.jax.org/external/festing/mouse/docs/C57BL.shtml). It is currently unclear, however, why some of the CR females remained infertile after the resumption of AL feeding at 15.5 months of age, but this may simply reflect individual variation in response to the CR protocol. Nevertheless, these results indicate that a delay of female reproductive senescence can be achieved using a procedure in adults that does not cause a delay in the timing of sexual maturation (i.e. CR initiated at weaning: Ball *et al*., 1947; Holehan & Merry, 1985; Gonzales *et al*., 2004) or require the use of genetic manipulation (e.g. *Bax* gene knockout: Perez *et al*., 1999, 2007).

Another observation made herein is that the ability of adult-onset CR to sustain function of the female reproductive axis with age appears to be mediated, at least in part, via maintenance of the ovarian follicle reserve. Although a systematic assessment of follicle dynamics throughout the entire study period (4–23 months of age) was not performed, the increased number of primordial follicles detected in aging females 4 months after the initiation of CR may reflect a decrease in follicle loss via atresia, reduced rates of primordial follicle growth activation or increased primordial follicle renewal, the latter of which has recently been shown to occur in adult female rodents (Johnson *et al*., 2004, 2005; Kerr *et al*., 2006; Bukovsky *et al*., 2007; Lee *et al*., 2007; Tilly & Rueda, 2008). Irrespective of how the follicle reserve is maintained by adult-onset CR, the quality of the oocytes remaining in the ovaries of the CR females does not appear to be compromised by advanced maternal age. In fact, our data show that both litter size and postnatal offspring survival rates were remarkably high for 15.5- to 23-month-old CR-then-AL-fed females, especially when compared with the same outcomes in considerably younger (10- to 15.5-month-old) continuously AL-fed controls. These findings offer compelling evidence that age-related deterioration of egg quality, which is widely believed to be a principal driving force behind poor pregnancy success rates in aging females (Navot *et al*., 1991), can be overcome by a dietary modification initiated in adult life. This conclusion aligns well with recent data from studies of domestic farm animals, which indicate that oocyte and embryo quality can be improved by a short-term reduction in feed intake (Lozano *et al*., 2003; Freret *et al*., 2006).

In summary, we have shown in mice that moderate CR initiated in adult life can dramatically extend function of the female reproductive axis into advanced age, with significant benefits noted not just in the females placed under CR but also in the survival rates of the offspring conceived by aged CR females once returned to an AL diet. Although these data suggest that there may be ways to safely extend reproductive function in aging females without negatively affecting adolescent growth or the timing of sexual maturation, it remains unknown if aging humans and nonhuman primates would respond similarly. Nevertheless, we are encouraged by an increasing body of evidence indicating that CR produces many of the same physiological, metabolic and hormonal adaptations in aging nonhuman primates (Roth *et al*., 2000, 2004; Bodkin *et al*., 2003; Ingram *et al*., 2006) and humans (Walford *et al*., 2002) that are observed in rodents. Moreover, in light of recent data from mice demonstrating the beneficial effects of prolonged ovarian function on the female body with age (Perez *et al*., 2007), sustaining the ovarian follicle pool by adult-onset CR may, irrespective of the fertility issues discussed herein, play a significant role in improving the overall quality of life of aging females normally compromised by endocrine changes associated with the loss of cyclic ovarian function. Thus, even if direct application of adult-onset CR protocols in humans is not feasible for the purpose of sustaining function of the female reproductive axis with age for fertility or health reasons, ongoing efforts to develop clinically relevant small molecule CR mimetics (Ingram *et al*., 2004, 2007; Baur & Sinclair, 2006; Austad, 2007; Chen & Guarente, 2007) may be of considerable interest to this important aspect of the biology of aging.

## Experimental procedures

### Animals

Virgin C57BL/6 female mice were obtained from the National Institute on Aging (NIA, Bethesda, MD, USA). Wild-type C57BL/6 adult male mice were obtained from Jackson Laboratories (Bar Harbor, ME, USA). All animal husbandry and experimental procedures were reviewed and approved by the institutional animal care and use committee of Massachusetts General Hospital.

### Feeding regimen

For these experiments, we used an adult-onset CR protocol developed by the NIA for their Biomarkers of Aging study (Turturro *et al*., 1999), in which CR is initiated at 14 weeks of age at 10% restriction, increased to 25% restriction at 15 weeks, and to 40% restriction at 16 weeks where it is maintained until the resumption (if so desired) of AL feeding at some point later in life (see http://www.nia.nih.gov/ResearchInformation/ScientificResources/AgedRodentColoniesHandbook/CaloricRestrictedColony.htm). Each female was housed individually in a conventional (nonventilated) cage. The CR females were fed once daily between 06:00 and 09:00 hours with 2.7 g (4.33 Kcal g^−1^) of NIH-31/NIA-fortified diet that provides the same levels of micronutrients as the NIH31 standard open-formula food used for AL feeding. The CR protocol was continued until the females reached 15.5 months of age, at which time the previously CR females were allowed AL access to NIH31 standard open-formula food. Water was provided AL for all animals during the study period. Mice were weighed weekly to assure proper effectiveness of the feeding regimen.

### Fertility testing

Mating trials were initiated at 10 months of age for all mice. For mating attempts during CR, a male mouse of proven fertility was housed overnight in a cage with a female and removed the following morning, so that the female could be fed her dietary food ration. The AL and CR females were handled identically. Upon return of CR females to AL feeding at 15.5 months of age, male mice were left with the females until obvious signs of pregnancy were observed, and then removed until the next mating attempt. Males were randomly rotated among the cages during the mating trials. The total number of offspring delivered per litter and the number of offspring delivered that were viable and survived were recorded separately for each pregnancy. Offspring that did not survive were either found dead at birth or died very shortly after delivery. All viable offspring were allowed to remain with the dam until weaning (day 21 postpartum), at which time the offspring were removed from the cages to allow for a subsequent mating attempt with the dam. For all offspring that survived, no overt anatomical or health complications were observed (data not shown).

### Ovarian follicle counts

Oocyte-containing follicles were scored by histomorphometric evaluation as originally described (Jones & Krohn, 1961), with slight modifications detailed previously (Tilly, 2003; Skaznik-Wikiel *et al*., 2007). Each ovary was given a numerical code so that all counts were conducted without knowledge of sample identity. Slides were then decoded and the total number of healthy and atretic immature follicles per ovary was calculated. Accuracy and reproducibility of the counts were independently confirmed by having a different observer reassess follicle numbers in randomly selected slides without knowledge of sample identity.

### Data presentation and analysis

Graphs depict results from each individual mouse or combined data obtained from independently replicated experiments (mean ± SEM). Where appropriate, the combined data were analyzed by a one-way analysis of variance followed by two-tailed *t*-test for statistical comparisons between mean values.

## References

[b1] Austad SN (2007). Vertebrate aging research 2006. Aging Cell.

[b2] Ball ZB, Barnes RH, Visscher MB (1947). The effects of dietary caloric restriction on maturity and senescence, with particular reference to fertility and longevity. Am. J. Physiol.

[b3] Baur JA, Sinclair DA (2006). Therapeutic potential of resveratrol: the *in vivo* evidence. Nat. Rev. Drug Discov.

[b4] Berg BN (1960). Nutrition and longevity in the rat. I. Food intake in relation to size, health and fertility. J. Nutr.

[b5] Bodkin NL, Alexander TM, Ortmeyer HK, Johnson E, Hansen BC (2003). Mortality and morbidity in laboratory-maintained rhesus monkeys and effects of long-term dietary restriction. J. Gerontol. A Biol. Sci. Med. Sci.

[b6] Buckler H (2005). The menopause transition: endocrine changes and clinical symptoms. J. Br. Menopause Soc.

[b7] Bukovsky A, Ayala ME, Dominguez R, Svetlikova M, Selleck-White R (2007). Bone marrow derived cells and alternative pathways of oogenesis in adult rodents. Cell Cycle.

[b8] Cargill SL, Carey JR, Muller HG, Anderson G (2003). Age of ovary determines remaining life expectancy in old ovariectomized mice. Aging Cell.

[b9] Charleston JS, Hansen KR, Thyer AC, Charleston LB, Gougeon A, Siebert JR, Soules MR, Klein NA (2007). Estimating human ovarian non-growing follicle number: the application of modern stereology techniques to an old problem. Hum. Reprod.

[b10] Chen D, Guarente L (2007). SIR2: a potential target for calorie restriction mimetics. Trends Mol. Med.

[b11] Faddy MJ, Gosden RG, Gougeon A, Richardson SJ, Nelson JF (1992). Accelerated disappearance of ovarian follicles in mid-life: implications for forecasting menopause. Hum. Reprod.

[b12] Freret S, Grimard B, Ponter AA, Joly C, Ponsart C, Humblot P (2006). Reduction of body-weight gain enhances in vitro embryo production in overfed superovulated dairy heifers. Reproduction.

[b13] Gonzales C, Voirol MJ, Giacomini M, Gaillard RC, Pedrazzini T, Pralong FP (2004). The neuropeptide Y Y1 receptor mediates NPY-induced inhibition of the gonadotrope axis under poor metabolic conditions. FASEB J.

[b14] Gosden RG, Laing SC, Felicio LS, Nelson JF, Finch CE (1983). Imminent oocyte exhaustion and reduced follicular recruitment mark the transition to acyclicity in aging C57BL/6J mice. Biol. Reprod.

[b15] Hamilton BE, Martin JA, Ventura SJ (2007). Births: preliminary data for 2006. Natl Vital Stat. Report.

[b16] Hansen KR, Knowlton NS, Thyer AC, Charleston JS, Soules MR, Klein NA (2008). A new model of reproductive aging: the decline in ovarian non-growing follicle number from birth to menopause. Hum. Reprod..

[b17] Holehan AM, Merry BJ (1985). The control of puberty in the dietary restricted female rat. Mech. Ageing Dev.

[b18] Ingram DK, Anson RM, de Cabo R, Mamczarz J, Zhu M, Mattison J, Lane MA, Roth GS (2004). Development of calorie restriction mimetics as a prolongevity strategy. Ann. N. Y. Acad. Sci.

[b19] Ingram DK, Roth GS, Lane MA, Ottinger MA, Zou S, de Cabo R, Mattison JA (2006). The potential for dietary restriction to increase longevity in humans: extrapolation from monkey studies. Biogerontology.

[b20] Ingram DK, Zhu M, Mamczarz J, Zou S, Lane MA, Roth GS, de Cabo R (2007). Calorie restriction mimetics: an emerging research field. Aging Cell.

[b21] Johnson J, Canning J, Kaneko T, Pru JK, Tilly JL (2004). Germline stem cells and follicular renewal in the postnatal mammalian ovary. Nature.

[b22] Johnson J, Bagley J, Skaznik-Wikiel M, Lee H-J, Adams GB, Niikura Y, Tschudy KS, Tilly JC, Cortes ML, Forkert R, Spitzer T, Iacomini J, Scadden DT, Tilly JL (2005). Oocyte generation in adult mammalian ovaries by putative germ cells in bone marrow and peripheral blood. Cell.

[b23] Jones PB, Krohn PL (1961). The relationships between age, numbers of oocytes and fertility in virgin and multiparous mice. J. Endocrinol.

[b24] Kerr JB, Myers M, Britt KL, Mladenovska T, Findlay JK (2006). Quantification of healthy follicles in the neonatal and adult mouse ovary: evidence for maintenance of primordial follicle supply. Reproduction.

[b25] van Kooij RJ, Looman CW, Habbema JD, Dorland M, te Velde ER (1996). Age-dependent decrease in embryo implantation rate after *in vitro* fertilization. Fertil. Steril.

[b26] Lee H-J, Sakamoto H, Luo H, Skaznik-Wikiel ME, Friel A, Niikura T, Tilly JC, Klein R, Styer A, Zuckerberg L, Tilly JL, Rueda BR (2007). Loss of CABLES1, a cyclin-dependent kinase-interacting protein that inhibits cell cycle progression, results in germline expansion at the expense of oocyte quality in adult female mice. Cell Cycle.

[b27] Lintern-Moore S, Everitt AV (1978). The effect of restricted food intake on the size and composition of the ovarian follicle population in the Wistar rat. Biol. Reprod.

[b28] Lozano JM, Lonergan P, Boland MP, O’Callaghan D (2003). Influence of nutrition on the effectiveness of superovulation programmes in ewes: effect on oocyte quality and post-fertilization development. Reproduction.

[b29] Martin B, Golden E, Carlson OD, Egan JM, Mattson MP, Maudsley S (2008). Caloric restriction: impact upon pituitary function and reproduction. Ageing Res. Rev.

[b30] Masoro EJ (2005). Overview of caloric restriction and ageing. Mech. Ageing Dev.

[b31] Merry BJ, Holehan AM (1979). Onset of puberty and duration of fertility in rats fed a restricted diet. J. Reprod. Fertil.

[b32] Navot D, Bergh PA, Williams MA, Garrisi GJ, Guzman I, Sandler B, Grunfeld L (1991). Poor oocyte quality rather than implantation failure as a cause of age-related decline in female fertility. Lancet.

[b33] Nelson JF, Gosden RG, Felicio LS (1985). Effect of dietary restriction on estrous cyclicity and follicular reserves in aging C57BL/6J mice. Biol. Reprod.

[b34] Osborne TB, Mendel LB, Ferry EL (1917). The effect of retardation of growth upon the breeding period and duration of life of rats. Science.

[b35] Perez GI, Robles R, Knudson CM, Flaws JA, Korsmeyer SJ, Tilly JL (1999). Prolongation of ovarian lifespan into advanced chronological age by *Bax*-deficiency. Nat. Genet.

[b36] Perez GI, Jurisicova A, Wise L, Lipina T, Kanisek M, Bechard A, Takai Y, Hunt P, Roder J, Grynpas M, Tilly JL (2007). Absence of the proapoptotic Bax protein extends fertility and alleviates age-related health complications in female mice. Proc. Natl Acad. Sci. USA.

[b37] Richardson SJ, Senikas V, Nelson JF (1987). Follicular depletion during the menopausal transition: evidence for accelerated loss and ultimate exhaustion. J. Clin. Endocrinol. Metab.

[b38] Roth GS, Ingram DK, Black A, Lane MA (2000). Effects of reduced energy intake on the biology of aging: the primate model. Eur. J. Clin. Nutr.

[b39] Roth GS, Mattison JA, Ottinger MA, Chachich ME, Lane MA, Ingram DK (2004). Aging in rhesus monkeys: relevance to human health interventions. Science.

[b40] Skaznik-Wikiel M, Tilly JC, Lee H-J, Niikura Y, Kaneko-Tarui T, Johnson J, Tilly JL (2007). Serious doubts over ‘Eggs Forever?’. Differentiation.

[b41] Tilly JL (2001). Commuting the death sentence: how oocytes strive to survive. Nat. Rev. Mol. Cell Biol.

[b42] Tilly JL (2003). Ovarian follicle counts – not as simple as 1, 2, 3. Reprod. Biol. Endocrinol.

[b43] Tilly JL, Rueda BR (2008). Stem cell contribution to ovarian development, function and disease. Endocrinology.

[b44] Turturro A, Witt WW, Lewis S, Haas BS, Lipman RD, Hart RW (1999). Growth curves and survival characteristics of the animals used in the Biomarkers of Aging program. J. Gerontol. A Biol. Sci. Med. Sci.

[b45] Ventura SJ (1989). Trends and variations in first births to older women, United States, 1970–86. Vital. Health Stat. 21.

[b46] Ventura SJ, Abma JC, Mosher WD, Henshaw S (2004). Estimated pregnancy rates for the United States, 1990–2000: an update. Natl. Vital Stat. Report.

[b47] Visscher MB, King JT, Lee CP (1952). Further studies on influence of age and diet upon reproductive senescence in strain A female mice. Am. J. Physiol.

[b48] Walford RL, Mock D, Verdery R, MacCallum T (2002). Calorie restriction in biosphere 2: alterations in physiologic, hematologic, hormonal, and biochemical parameters in humans restricted for a 2-year period. J. Gerontol. A. Biol. Sci. Med. Sci.

[b49] Wu JM, Zelinski MB, Ingram DK, Ottinger MA (2005). Ovarian aging and menopause: current theories, hypotheses, and research models. Exp. Biol. Med. (Maywood).

